# Serotonin Modulates AhR Activation by Interfering with CYP1A1-Mediated Clearance of AhR Ligands

**DOI:** 10.33594/000000209

**Published:** 2020-02-05

**Authors:** Christopher R. Manzella, Max Ackerman, Megha Singhal, Alexander L. Ticho, Justin Ceh, Waddah A. Alrefai, Seema Saksena, Pradeep K. Dudeja, Ravinder K. Gill

**Affiliations:** a Department of Physiology & Biophysics, University of Illinois at Chicago, Chicago, IL, USA,; b Division of Gastroenterology & Hepatology, University of Illinois at Chicago, Chicago, IL, USA,; c Jesse Brown VA Medical Center, Chicago, IL, USA

**Keywords:** Serotonin (5-HT), Cytochrome P450 1A1 (CYP1A1), Aryl hydrocarbon receptor (AhR)

## Abstract

**Background/Aims:**

Serotonin (5-hydroxytryptamine, 5-HT) is a neurotransmitter and hormone with important physiological functions in many organs, including the intestine. We have previously shown that 5-HT activates the aryl hydrocarbon receptor (AhR) in intestinal epithelial cells (IECs) via a serotonin transporter (SERT)-dependent mechanism. AhR is a nuclear receptor that binds a variety of molecules including tryptophan (TRP) metabolites to regulate physiological processes in the intestine including xenobiotic detoxification and immune modulation. We hypothesized that 5-HT activates AhR indirectly by interfering with metabolic clearance of AhR ligands by cytochrome P450 1A1 (CYP1A1).

**Methods:**

Inhibition of CYP1A1 activity by 5-HT was assessed in the human intestinal epithelial cell line Caco-2 and recombinant CYP1A1 microsomes using both luciferase and LC-MS/MS. Degradation of 5-HT by recombinant CYP1A1 was measured by LC-MS/MS. For *in vitro* studies, CYP1A1 and CYP1B1 mRNA expression levels were measured by RT-PCR and CYP1A1 activity was measured by ethoxyresorufin-O-deethylase (EROD) assays. For *in vivo* studies, AhR ligands were administered to SERT KO mice and WT littermates and intestinal mucosa CYP1A1 mRNA was measured.

**Results:**

We show that 5-HT inhibits metabolism of both the pro-luciferin CYP1A1 substrate Luc-CEE as well as the high affinity AhR ligand 6-formylindolo[3,2-*b*] carbazole (FICZ). Recombinant CYP1A1 assays revealed that 5-HT is metabolized by CYP1A1 in an NADPH dependent manner. Treatment with 5-HT in TRP-free medium, which is devoid of trace AhR ligands, showed that 5-HT requires the presence of AhR ligands to activate AhR. Cotreatment with 5-HT and FICZ confirmed that 5-HT potentiates induction of AhR target genes by AhR ligands. However, this was only true for ligands which are CYP1A1 substrates such as FICZ. Administration of β-napthoflavone by gavage or indole-3-carbinol via diet to SERT KO mice revealed that lack of SERT impairs intestinal AhR activation.

**Conclusion:**

Our studies provide novel evidence of crosstalk between serotonergic and AhR signaling where 5-HT can influence the ability of AhR ligands to activate the receptor in the intestine.

## Introduction

The gastrointestinal (GI) tract is an important site of interface between the host and the environment. Of the many mechanisms that have evolved to respond to environmental stimuli at this barrier, the nuclear receptor aryl hydrocarbon receptor (AhR) has been recognized as one of the most crucial, as it binds a diverse array of ligands to serve a variety of physiological functions [1]. The prototypical gene targets for AhR are the cytochrome P450 family 1 (CYP1) enzymes including CYP1A1 [2]. Traditionally studied for its role in recognizing xenobiotics such as polycyclic aromatic hydrocarbons (PAHs) and facilitating their metabolism and elimination, AhR is now recognized to play important roles in cell proliferation and survival, as well as mucosal immune homeostasis [3]. In fact, AhR activation has been shown to promote mucosal healing and decrease intestinal inflammation in animal models of inflammatory bowel disease (IBD] including both DSS and TNBS-induced colitis [4–7]. Conversely, knockout of AhR specifically in intestinal epithelial cells has deleterious consequences on the outcome of experimental colitis [8].

AhR has evolved to recognize and bind a variety of molecules from both exogenous and endogenous sources. Benzo[a]pyrene and 3-methylcholranthene are environmental PAHs which were among the first ligands studied to bind AhR [9]. Another class of exogenous ligands are diet-derived ligands, which primarily consist of TRP-derived phytochemicals [10]. Most vegetables contain high levels of a TRP derivative called glucobrassicin, which is cleaved into the weak AhR ligand indole-3-carbinol (I3C) during mastication by plant-derived myrosinase enzymes [11]. I3C is converted to high affinity AhR ligands including 3, 3’-diindoylmethane (DIM] and indole [3, 2-*b*]carbazole (ICZ) as a result of exposure to the acidic environment of the stomach. 6-formylindolo[3, 2-*b*]carbazole (FICZ), which is very similar in structure and AhR binding affinity to ICZ, is proposed to be the major endogenous ligand for AhR [12]. Other endogenous ligands include both bacteria-derived indoles and condensation products of the TRP-derived metabolite kynurenine [13, 14].

**Fig. 1.**
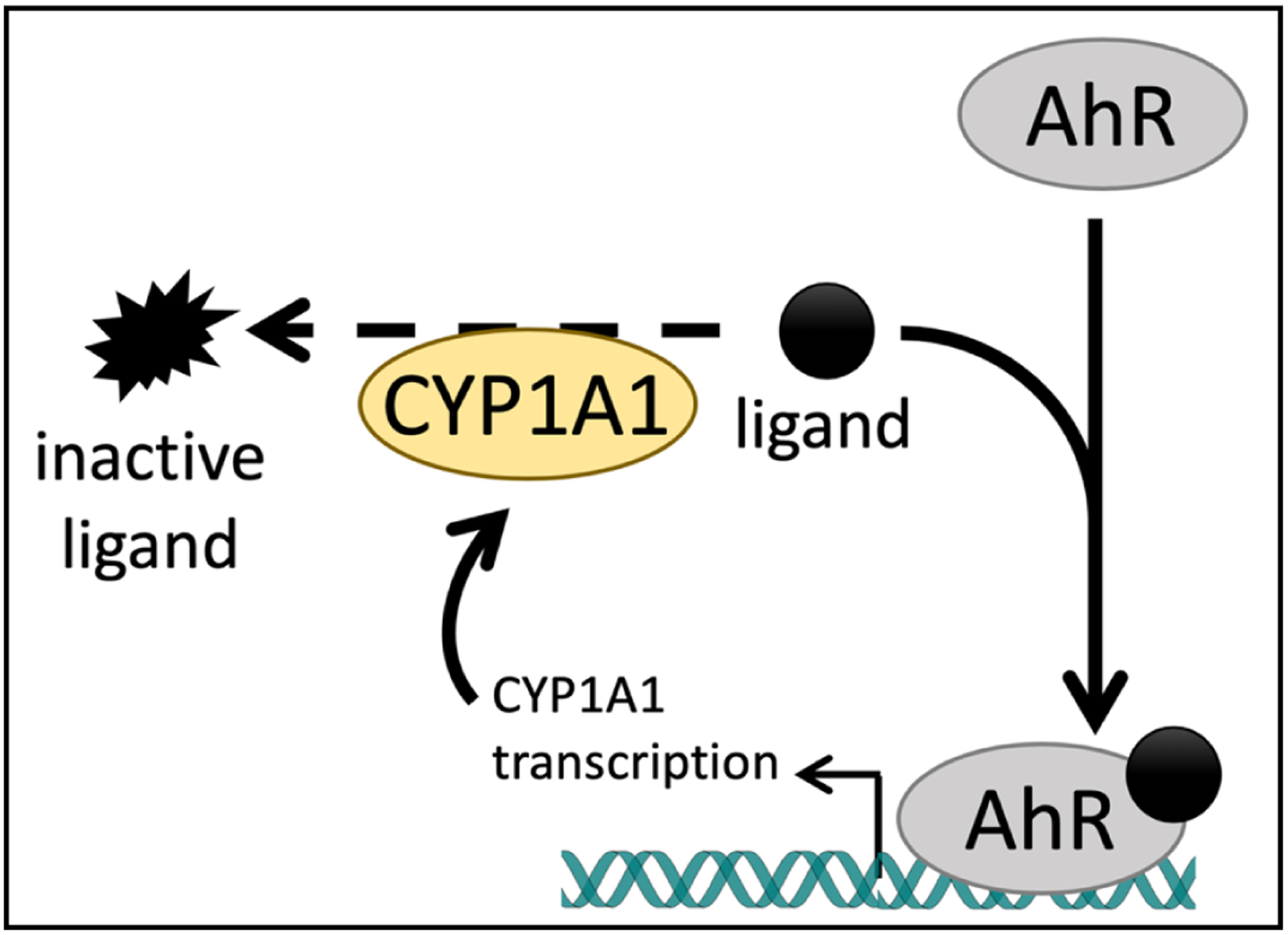
Ligand degradation by CYP1A1 as a negative feedback mechanism for AhR signaling. After a ligand binds AhR, AhR translocates to the nucleus and induces transcription of CYP1A1 mRNA and an increase in CYP1A1 enzymatic activity. This increased CYP1A1 degrades AhR ligands into inactive metabolites in order to terminate AhR signaling [15, 16].

The AhR signaling pathway is tightly controlled by an intrinsic negative feedback mechanism. More precisely, AhR ligands facilitate their own degradation by inducing expression and activity of the AhR gene target CYP1A1 (Fig. 1) [15, 16]. In accordance with this mechanism, FICZ is both a high-affinity AhR ligand as well as an excellent substrate for CYP1A1 [17]. Overexpression of CYP1A1 leads to increased AhR ligand degradation and reduced AhR activity [16]. Conversely, inhibition of CYP1A1 activity leads to decreased ligand inactivation and heightened activation of the AhR pathway [15]. This has fueled the hypothesis that there may be endogenous inhibitors of CYP1A1 enzymatic activity that could increase AhR activation by preventing the degradation of its ligands.

We have recently shown that serotonin (5-hydroxytryptamine, 5-HT) is able to activate AhR [18]. 5-HT is a TRP-derived neurotransmitter and hormone that has diverse physiological roles in several body systems including the nervous system and the GI tract. In the gut, 5-HT promotes motility [19], modulates electrolyte and fluid transport [20–22], and participates in neural reflexes [23, 24]. Activation of AhR by 5-HT is independent of the membrane-bound family of 5-HT receptors, but rather is dependent upon intracellular transport via the plasma membrane 5-HT transporter (SERT) [18]. In the gut mucosa, SERT functions to regulate the extracellular availability of 5-HT by facilitating high-affinity transport into enterocytes and other cell types [25]. Once inside the cell, intracellular monoamine oxidases are able to act upon 5-HT to facilitate conversion to 5-hydroxyindole-3-acetic acid (5-HIAA] [26]. Dysregulation of the 5-HT system has been demonstrated to play a role in the pathophysiology of gut disorders including IBD. Interestingly, expression of mucosal SERT is decreased in experimental colitis [27, 28] as well as in IBD patients [29–32]. Conversely, SERT KO mice which lack high affinity transport of 5-HT into the intracellular compartment are more susceptible to intestinal inflammation [33, 34].

While we have previously established 5-HT as an endogenous activator of AhR, the precise mechanism of 5-HT-dependent AhR activation was unclear. Here, we report that 5-HT activates AhR indirectly via inhibition of CYP1A1 enzymatic clearance of AhR ligands using cell-free models and the human intestinal epithelial cell line Caco-2. Further, we show that SERT KO mice have an impaired response to exogenously administered AhR ligands. Our findings support a new role for gut-derived 5-HT as a key regulator of AhR ligand availability and receptor activation.

## Materials and Methods

### Materials

Serotonin (5-HT] hydrochloride, indole, 3-methylindole (3-MI], tryptamine hydrochloride (TRYPT), α-naphthoflavone (α-NF), β-naphthoflavone (β-NF), 5-hydroxyindoleacetic acid (5-HIAA), L-tryptophan (TRP), indole-3-carbinol (I3C), 2,3,7, 8-Tetrachlorodibenzo-*p*-dioxin (TCDD), and indole-3-acetic acid (IAA) were obtained from Sigma. 6-formylindolo [3, 2*b*]carbazole (FICZ) was obtained from Tocris. 7-ethoxyresorufin and indolo [3, 2*b*]carbazole (ICZ) were obtained from Santa-Cruz. TRP was recrystallized from 70% boiling ethanol in water and the crystals were collected and washed in absolute ethanol under vacuum prior to use in order to remove residual FICZ. Caco-2 cells, Eagle’s minimum essential medium (EMEM), and 0.25% trypsin-EDTA were obtained from American Type Culture Collection (ATCC). Fetal bovine serum (FBS) was purchased from Gibco. Penicillin and streptomycin were purchased from Invitrogen. Cell culture flasks, cell culture plates, CYP1A1 microsomes, negative control insect microsomes, and 1X phosphate buffered saline (PBS) were purchased from Corning. TRP-free medium as a custom formulation was purchased from AthenaES. LC-MS vials, LC column, and LC column guards were purchased from Phenomenex. All solvents used for LC-MS/MS were of LC-MS grade and were purchased from Fisher. TRIzol was also purchased from Fisher. NADPH regeneration system, luciferase assay, and CYP1A1 P450-Glo assay kits were purchased from Promega. The Bradford assay kit was purchased from Biorad. Brilliant SYBGR green qPCR master mix kit was purchased from Stratagene.

### Animals

SERT KO heterozygous mice were purchased from Jackson Laboratory and bred in-house. The mice were housed in autoclaved polypropylene cages with corncob bedding. The mice had free access to food (Teklad Irradiated LM-485 Mouse/Rat Diet 7912, Envigo) and water and a 12 h light/dark cycle. For β-NF experiments, 3-month old male WT and SERT KO littermates were administered 50 mg/kg β-NF in corn oil by gavage daily for 3 days prior to euthanasia. For I3C studies, 3-month old male and female mice were fed AIN-76A synthetic diet or AIN-76A with 200 ppm I3C for 3 weeks (Research Diets). After euthanasia, intestines were immediately resected and separated into jejunum, ileum, and colon. Mucosa was scraped and flash frozen in liquid nitrogen and stored at −80°C for RNA extraction and protein lysate preparation. Liver and lung were resected and immediately flash frozen in liquid nitrogen and stored at −80°C until RNA extraction. All animal studies were performed in accordance with institutional guidelines and regulations and as approved by the Animal Care Committees of the University of Illinois at Chicago and the Jesse Brown Veterans Affairs Medical Center.

### Cell Culture

Caco-2 cells were grown routinely in EMEM supplemented with 10%. All cell culture medium used for maintenance contained 100 IU/mL penicillin and 100 μg/mL streptomycin. Caco-2 cells were maintained in 5% CO_2_−95% air at 37°C. Cells were maintained in 75 cm^2^ or 150 cm^2^ flasks and were split using 0.25% trypsin-EDTA. In preparation for treatment and uptake experiments, Caco-2 cells were plated on 24-well plates on plastic supports at a density of 2 × 10^4^ cells/well. Caco-2 cells were maintained 10–14 d post-plating to allow differentiation before treatments were performed for all experiments unless indicated. Cells between passages 25 and 45 were used for all experiments. All treatments were given in serum-free EMEM unless otherwise indicated. All cells treated in TRP-free medium were switched from complete medium to TRP-free medium for 1 h prior to the start of treatment. All solutions containing 5-HT, ICZ, or FICZ were protected from light.

### RNA Extraction and Real Time RT-PCR

RNA was extracted from cells or mouse tissue using TRIzol according to the manufacturer’s instructions. Quantitative RT-PCR was performed using SYBR Green fluorescence by reverse-transcribing and amplifying equal amounts of RNA using the Brilliant SYBGR green qPCR master mix kit. The gene-specific primer sequences are listed in Supplementary Table S1 (for all supplemental material see www.cellphysiolbiochem.com). The relative mRNA levels were normalized to *GAPDH* mRNA levels using the ΔCt method.

### Ethoxyresorufin-O-deethylase (EROD) Assay

The CYP1A1-dependent ethoxyresorufin-O-dethylase (EROD) activity of Caco-2 cells was assayed by first treating the cells with 5-HT for FICZ in serum-free media for the indicated time point. The treatment media was removed and washed with 1X PBS before 300 μl of 50 mM NaHPO_4_ pH 8.0 containing 2 mM 7-ethoxyresorufin was added to each well of a 24-well plate. The cells were incubated at 37°C for 20 min before termination of the reaction by removal of the medium. Medium was transferred to a 96-well plate and formation of resorufin was quantified on a multiwell plate reader in triplicate with the excitation/ emission wavelengths of 544/590. The activity was expressed relative to the amount of protein present as determined by Bradford assay according to the manufacturer’s protocol.

### P450-Glo CYP1A1 Activity Assay

For cell-based assays, Caco-2 cells were plated at low density and allowed to differentiate in 24-well plates as described above. Cells were pretreated for 30 min in serum-free EMEM with vehicle, 5-HT, or α-NF for 30 min before incubation with each test compound along with 50 μM Luc-CEE pro-luciferin CYP1A1 substrate for 3 h. Media was collected and combined with an equal volume of luciferin detection reagent and incubated at room temperature for 20 min. Luminescence was recorded using a single tube luminometer (Promega), and a no-cell control was subtracted from each measurement to account for background. Relative CYP1A1 activity was taken as the luminescence after incubation of each test compound divided by the luminescence after incubation with vehicle. The activity was expressed relative to the amount of protein present as determined by Bradford assay according to the manufacturer’s protocol.

For cell-free assays, microsomes containing recombinant human CYP1A1 expressed in baculovirus infected insect cells (BTI-TN-5B1–4) were used. Reactions (final volume 50 ml) were performed in triplicate and contained 0.5 pmol CYP1A1, 100 mM KPO_4_ (pH 7.4), 30 mM Luc-CEE, varying concentrations of test compounds ranging from 1 M to 1 mM, and NADPH regeneration system components 1.3 mM NADP+, 3.3 mM glucose-6-phosphate, 3.3 mM MgCl_2_, and 0.4 U/mL glucose-6-phosphate dehydrogenase. All components except the NADPH regeneration system were combined (25 μl) and pre-incubated at 37°C for 10 min in 1.5 mL tubes. The reactions were initiated by adding an equal volume of 2X NADPH regeneration system (25 μl, also pre-incubated at 37°C) and placed at 37°C for 10 min. Reactions were terminated by the addition of luciferin detection reagent (50 ml) and incubated at room temperature for 20 min. Luminescence was recorded using a single tube luminometer, and a control without NADPH was subtracted from each measurement to account for background. CYP1A1 activity was taken as the percentage of the luminescence after incubation with vehicle.

### FICZ Metabolism Assay

Caco-2 cells were split at low density (1:8) into 150 cm^2^ flasks and allowed to differentiate for two weeks. At the start of the assay, flasks were incubated with 5-HT (5 μM), α-NF (5 μM), or vehicle for 30 min. Next, cells were incubated with 50 nM FICZ along with test compounds for 30 min to allow for intracellular accumulation. FICZ-containing medium was removed, cells were washed with warm 1X PBS, and cells were incubated with test compounds for 2 h. Cells were washed with ice-cold 1X PBS, scraped in ice-cold 1X PBS (N_2_ purged), and centrifuged for 10 min at 500 G. 1X PBS was removed and 750 ml of cold acetonitrile (N_2_ purged) was added to each cell pellet. Cells were sonicated and centrifuged for 10 min at 10,000 G to pellet precipitated protein and the extract was added to a new tube. Extracts were evaporated under N_2_ and dried samples were analyzed immediately by LC-MS/MS.

### Recombinant CYP1A1 Reactions

Microsomes containing recombinant human CYP1A1 expressed in baculovirus infected insect cells (BTI-TN-5B1–4) and negative control microsomes isolated from baculovirus infected insect cells (BTI-TN-5B1–4) were used. Reactions (final volume 50 μl) were performed in duplicate and were carried out either in the presence or absence of NADPH. All reactions contained 2 pmol CYP1A1 or an equivalent amount of control microsomal protein, 100 mM KPO_4_ (pH 7.4), and 1 μM 5-HT. Reactions with NADPH also contained NADPH regeneration system components 1.3 mM NADP+, 3.3 mM glucose-6-phosphate, and, 0.4 U/mL glucose-6-phosphate dehydrogenase. Reactions without NADPH were supplemented with 3.3 mM MgCl_2_ instead. All components except the NADPH regeneration system or MgCl_2_ were combined (25 μl) and pre-incubated at 37°C for 10 min in 1.5 mL tubes. The reactions were initiated by adding an equal volume of 2X NADPH regeneration system or 6.6 mM MgCl_2_ (both 25 μl, also pre-incubated at 37°C) and placed at 37°C for the indicated time points. Reactions were terminated by adding 350 μl of ice-cold acetonitrile (N_2_ purged) and vortexing briefly. Samples were centrifuged at 10, 000 G for 5 min to pellet down microsomes and 350 μl of extract was transferred to a new tube. Extracts were evaporated under N_2_ and dried samples were stored at −80°C until analysis by LC-MS/MS.

### LC-MS/MS measurements

Samples were reconstituted in 100 μl water/acetonitrile/formic acid (99/1/0.1 (v/v)) (N_2_ purged), vortexed, and added to LC vials. Samples were measured by LC-MS/MS using an Agilent 1200 Series HPLC coupled to an AB Sciex QTRAP 5500. Samples (5 μl) were injected on a 100 × 2.1 mm Luna® Omega PS C18 column with 5-μm silica particles equipped with a 4 × 2.0 mm C18 guard column. The column temperature was set to 40°C. 5-HT, 5-HIAA, and TRP were separated using an increasing gradient of acetonitrile created by solvent A as water with 0.1% formic acid and solvent B as acetonitrile with 0.1% formic acid at a flow rate of 400 l/min with a 3 min equilibration at 97% solvent A prior to injection. FICZ was separated using the same solvent system with an increasing gradient from 50% solvent B to 100% solvent B at a flow rate of 400 μl/min with a 2.5 min equilibration at 50% solvent B prior to injection. 5-HT was measured using LC method 1 (Supplementary Table S2), TRP and 5-HIAA was measured using LC method 2 (Supplementary Table S3), and FICZ was measured using LC method 3 (Supplementary Table S4). For detection of 5-HT, 5-HIAA, and TRP, the mass spectrometer was operated in electrospray ionization positive mode (ESI+) with multiple reaction monitoring (MRM). For detection of FICZ, the mass spectrometer was operated in electrospray ionization negative mode (ESI−) with MRM. Parameters for MRM are given in Supplementary Table S5. The abundance of analyte was determined by measuring peak area.

### Statistics

For all experiments, 1-way or 2-way ANOVA followed by Tukey’s, Dunnett’s, or Bonferroni’s multiple comparisons test, or 2-tailed unpaired or paired Student’s *t*-test were utilized for statistical analysis as indicated with alpha set to 0.05. Values are means ± SEM and all experiments were performed in at least triplicate on ≥ 3 separate occasions. IC_50_ values were determined by plotting log[inhibitor] vs. luciferase activity and determining the concentration which gives half of the maximum activity, assuming a Hill slope of −1.0. *p < 0.05, **p < 0.01, ***p < 0.001, ****p < 0.0001 for comparisons as indicated. Analyses were performed using GraphPad Prism (Prism 8).

## Results

### 5-HT Interferes with CYP1A1 Activity

**Fig. 2.**
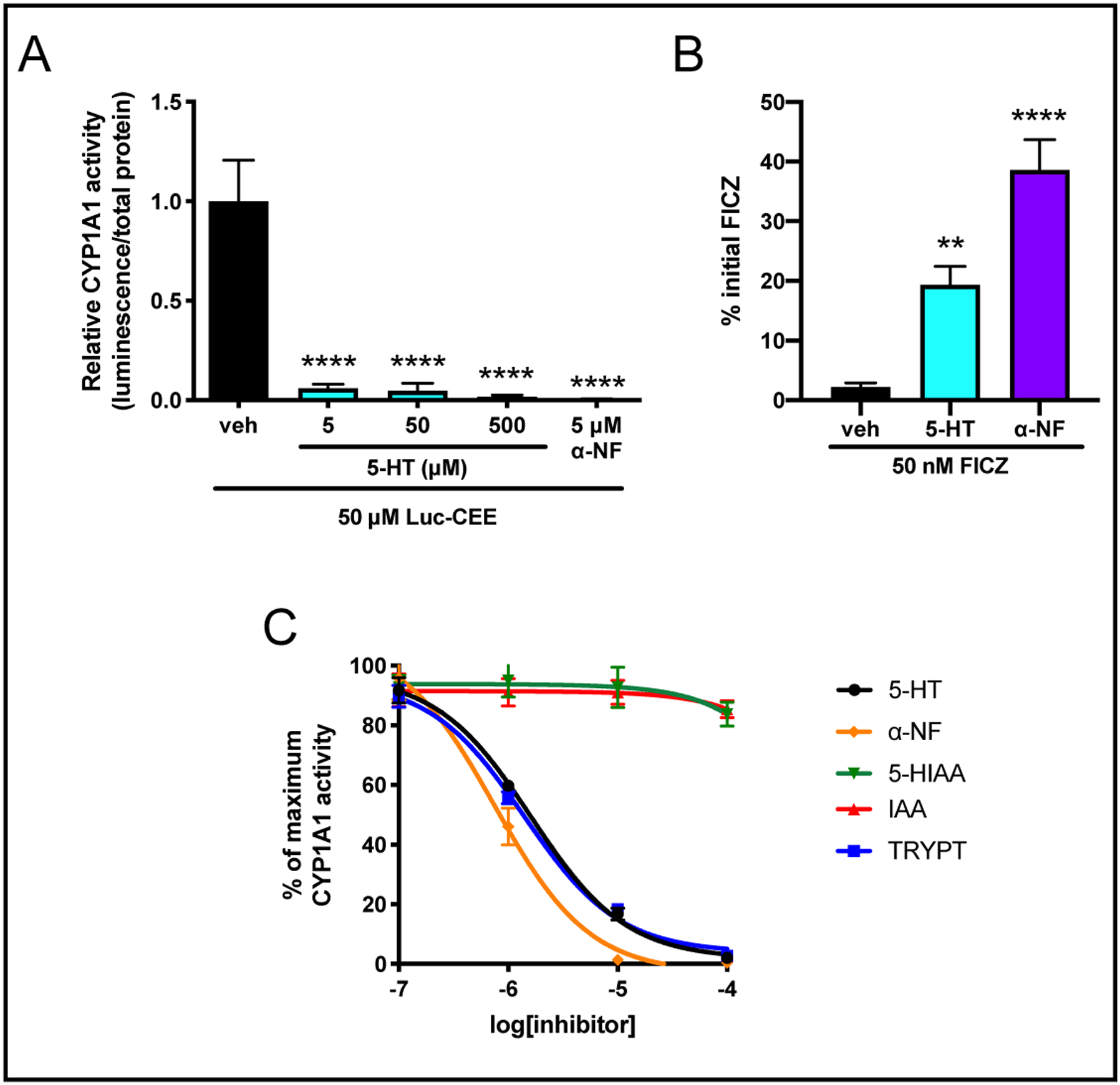
5-HT inhibits CYP1A1 activity. (A) Caco-2 cells were plated at low density and allowed to differentiate for 10–14 d in medium containing 10% serum before the assay was performed. Cells were co-incubated with 50 μM Luc-CEE and either vehicle (veh), 5-HT (5 – 500 μM), or α-NF (5 μM) for 3 h in serum-free medium before measurement of luciferase activity (n = 3–4). Data represent the relative CYP1A1 activity measured as luciferase activity detected in the medium after incubation relative to the vehicle treated cells. Assays were performed using triplicate wells for each treatment and values were normalized to total protein by Bradford assay. Data analyzed by 1-way ANOVA followed by Dunett’s multiple comparison’s test. ****P<0.0001 (B) Caco-2 cells were split at low density into 150 cm^2^ flasks and grown for 2 weeks before the assay was performed. Cells were loaded with 50 nM FICZ for 30 min and intracellular FICZ levels were measured by LC-MS/MS 2 h after removal of FICZ in the presence or absence of vehicle, 5-HT (5 μM), or α-NF (5 μM). Intracellular FICZ levels were normalized to intracellular TRP levels also determined by LC-MS/MS and values are expressed as a percentage of initial FICZ measured at 0 h (n = 3]. Data analyzed by 1-way ANOVA followed by Dunett’s multiple comparison’s test. **P<0.01, ****P<0.0001 vs. cells incubated with vehicle (C) NADPH-dependent metabolism of Luc-CEE (30 μM) by recombinant CYP1A1 (10 nM) was measured in presence or absence of α-NF or TRP metabolites 5-HT, tryptamine (TRYPT), 5-hydroxyindoleacetic acid (5-HIAA), or indole-3-acetic acid (IAA) (1 – 1000 μM) at 37°C for 10 min. Assays were performed in triplicate and values were normalized to activity in the presence of vehicle (n = 3).

We first investigated the potential of 5-HT to interfere with basal CYP1A1 enzymatic activity in model intestinal epithelial cell line Caco-2. When Caco-2 cells were co-incubated with the pro-luciferin CYP1A1 substrate Luc-CEE (50 μM] and increasing concentrations of 5-HT (5 – 500 μM], the measured basal CYP1A1 activity determined by Luc-CEE conversion to luciferin was dramatically decreased (>90% inhibition at 5 μM] (Fig. 2A). The compound α-naphthoflavone (α-NF], an established CYP1A1 inhibitor, was used as a positive control for CYP1A1 inhibition. At 5 μM, α-NF completely blocked basal CYP1A1 activity. The ability of 5-HT and α-NF to inhibit degradation of FICZ (50 nM) by Caco-2 cells was also assessed. In accordance with the luciferase-based assay, 5-HT (5 μM) or α-NF (5 μM) were able to prevent metabolism of FICZ (Fig. 2B).

In order to establish a direct effect of 5-HT on CYP1A1 enzymatic activity, a cell-free system consisting of recombinant CYP1A1 (10 nM) was used. 5-HT was able to inhibit metabolism of Luc-CEE (30 μM) in a concentration-dependent manner with an IC_50_ of 1.62 μM [95% CI interval, (1.12 μM, 2.34 μM)] for the assay (Fig. 2C). Tryptamine (TRYPT) inhibited CYP1A1 metabolism of Luc-CEE in a similar manner with an IC_50_ of 1.40 μM (0.95 μM, 2.06 μM), while α-NF inhibited Luc-CEE metabolism with an IC_50_ of 0.76 μM (0.41 μM, 1.41 μM). Interestingly, neither the 5-HT metabolite 5-hydroxyindoleacetic acid (5-HIAA) or the AhR ligand indole-3-acetic acid (IAA) inhibited CYP1A1 metabolism of Luc-CEE suggesting that the carboxylic acid moiety is incompatible with CYP1A1 binding. These data are consistent with the idea that 5-HT, and not its downstream metabolite 5-HIAA, is able to interfere with CYP1A1 activity.

### 5-HT is Degraded by CYP1A1 Enzymatic Activity

**Fig. 3.**
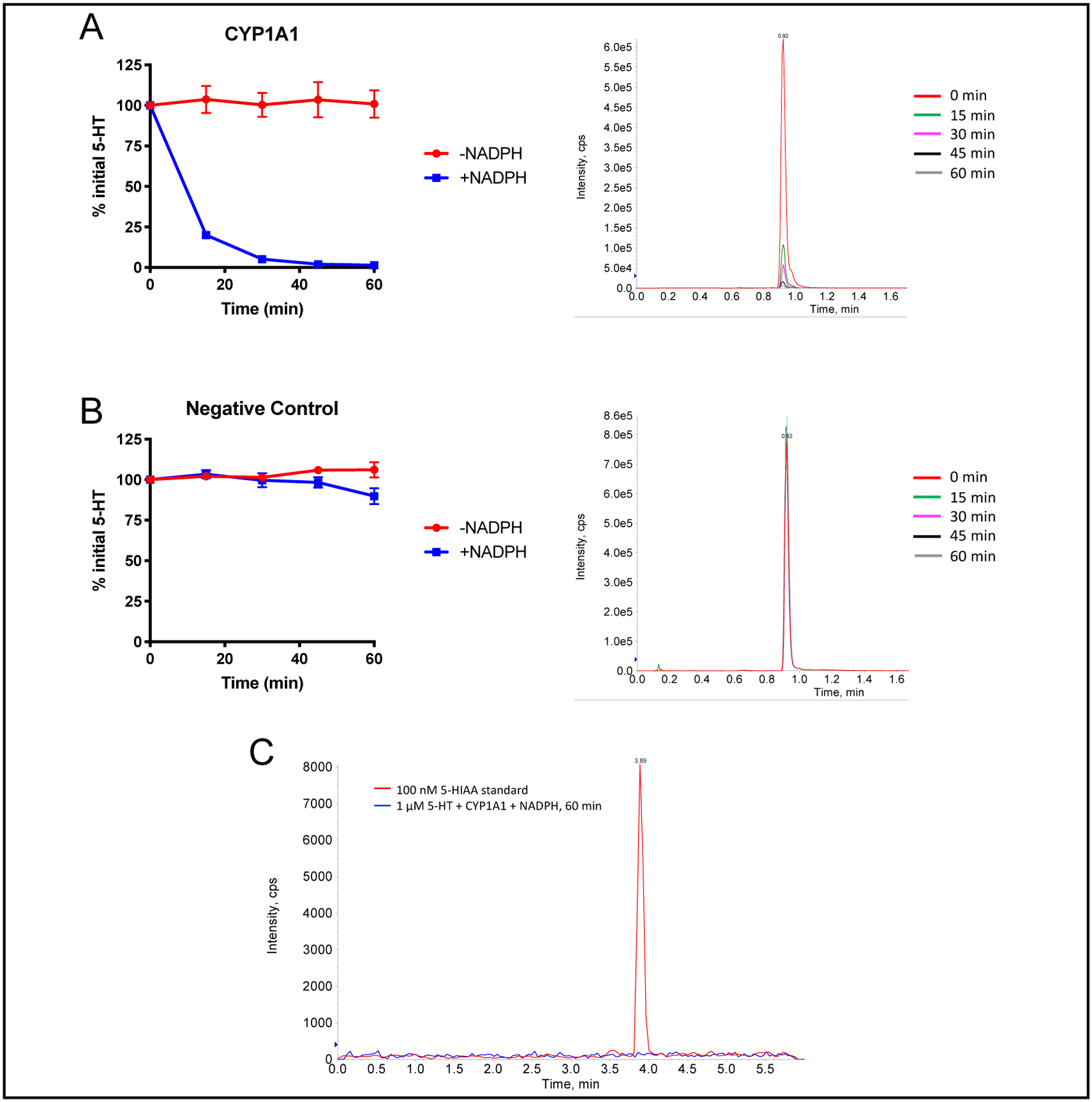
5-HT is metabolized by CYP1A1 and is not converted to 5-HIAA. Human recombinant CYP1A1 (40 nM) (A) or negative control microsomes (B) were incubated with 5-HT (1 μM) and 5-HT levels in the presence of absence of co-factor NADPH was measured by LC-MS/MS at the indicated time points (0 – 60 min). Data are shown as the percentage of 5-HT at the indicated time point relative to 0 min. Representative chromatograms showing the 5-HT peak over time in the presence of NADPH are also shown (n = 3). (C) 5-HIAA was measured in the reaction mixture after 60 min in the presence of NADPH (blue) and a 100 nM 5-HIAA standard was also measured (red).

To determine if interference of CYP1A1 activity by 5-HT is a consequence of 5-HT acting as a substrate for the enzyme or 5-HT being a non-substrate inhibitor, 5-HT (1 μM) was incubated with recombinant CYP1A1 (40 nM) and the relative abundance of 5-HT in the reaction mixture was measured by LC-MS/MS over 1 h. 5-HT rapidly disappeared from the reaction mixture with only ~20% of the initial 5-HT remaining after 15 min of incubation and nearly all 5-HT depleted from the reaction after 1 h of incubation (Fig. 3A). To confirm that 5-HT is not being metabolized by any native enzymes in the microsomal preparation, the assay was repeated using negative control microsomes. In contrast to the recombinant CYP1A1 microsomes, there was no significant NADPH-dependent metabolism of 5-HT in the control microsomes (Fig. 3B). Of note, no 5-HIAA was detected after 1 h of incubation with CYP1A1 in the presence of NADPH, confirming that 5-HT is not converted to 5-HIAA by CYP1A1 (Fig. 3C).

### 5-HT Requires the Presence of AhR Ligands to Induce CYP1A1

**Fig. 4.**
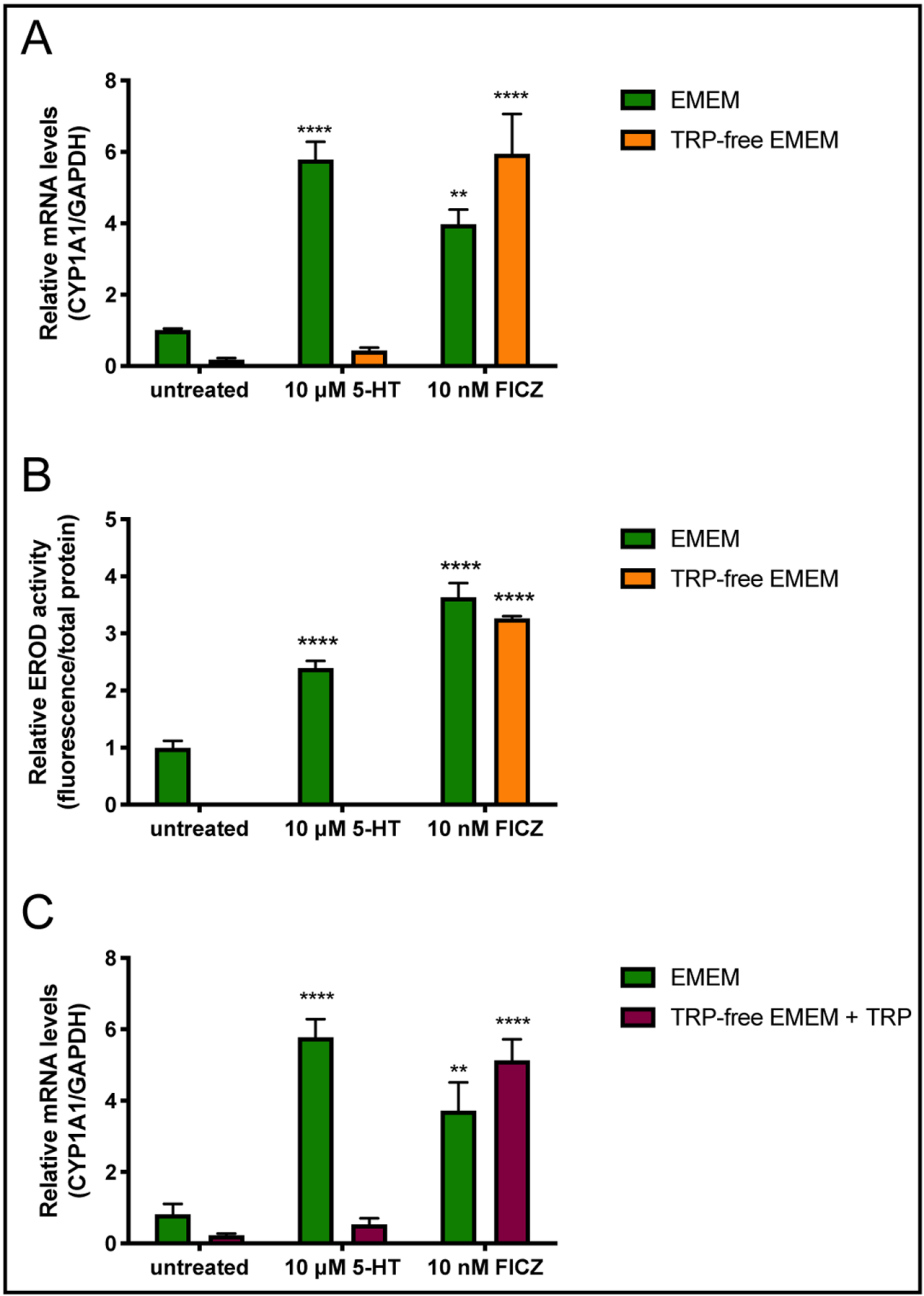
5-HT requires the presence of trace AhR ligands to induce CYP1A1 expression and activity. Caco-2 cells were plated at low density and allowed to differentiate for 10–14 d in medium containing 10% serum before treatments were performed. Cells were treated with 5-HT (10 μM) or FICZ (10 nM) for 8 h in EMEM or TRP-free EMEM. CYP1A1 mRNA (A) was quantified by qPCR and CYP1A1 activity (B) was measured by the ethoxyresorufin-O-deethylase (EROD) assay (n = 3). EROD assays were performed using triplicate wells for each treatment and values were normalized to total protein by Bradford assay. Results are expressed as fold-change mRNA or activity relative to untreated cells in EMEM. (C) The experiment was repeated except recrystallized TRP was added to the TRP-free media immediately prior to treatment. CYP1A1 mRNA was quantified by qPCR and results are expressed as fold-change mRNA relative to untreated cells in EMEM (n = 3). Data analyzed by 2-way ANOVA followed by Dunnett’s multiple comparison’s test. **P<0.01, ****P<0.0001 vs. untreated cells in the same media.

We have shown previously that 5-HT is able to induce CYP1A1 expression in commercial cell culture medium [18], which contains picomolar levels of FICZ formed from TRP in the medium, even when protected from light [15, 35]. In order to examine if 5-HT is able to induce CYP1A1 in the absence of trace AhR ligands, Caco-2 cells were treated with 5-HT (10 μM) in commercial Eagle’s Essential Minimum Medium (EMEM) or TRP-free EMEM for 8 h. 5-HT was able to induce *CYP1A1* mRNA (Fig. 4A) and activity as measured by EROD assay (Fig. 4B) in commercial medium, but did not induce CYP1A1 in TRP-free medium. Additionally, CYP1A1 induction by exogenously added FICZ (10 nM) was identical in both commercial and TRP-free medium. In order to rule out that the absence of TRP itself was responsible for this effect, the experiment was repeated with fresh recrystallized TRP added to the TRP-free medium immediately prior to use. The results of this experiment were identical to those when no TRP was added (Fig. 4C). These data strongly indicate that 5-HT requires the presence of trace AhR ligands in cell culture medium in order to induce CYP1A1.

### 5-HT Potentiates AhR Activation by CYP1A1-Substrate AhR Ligands

**Fig. 5.**
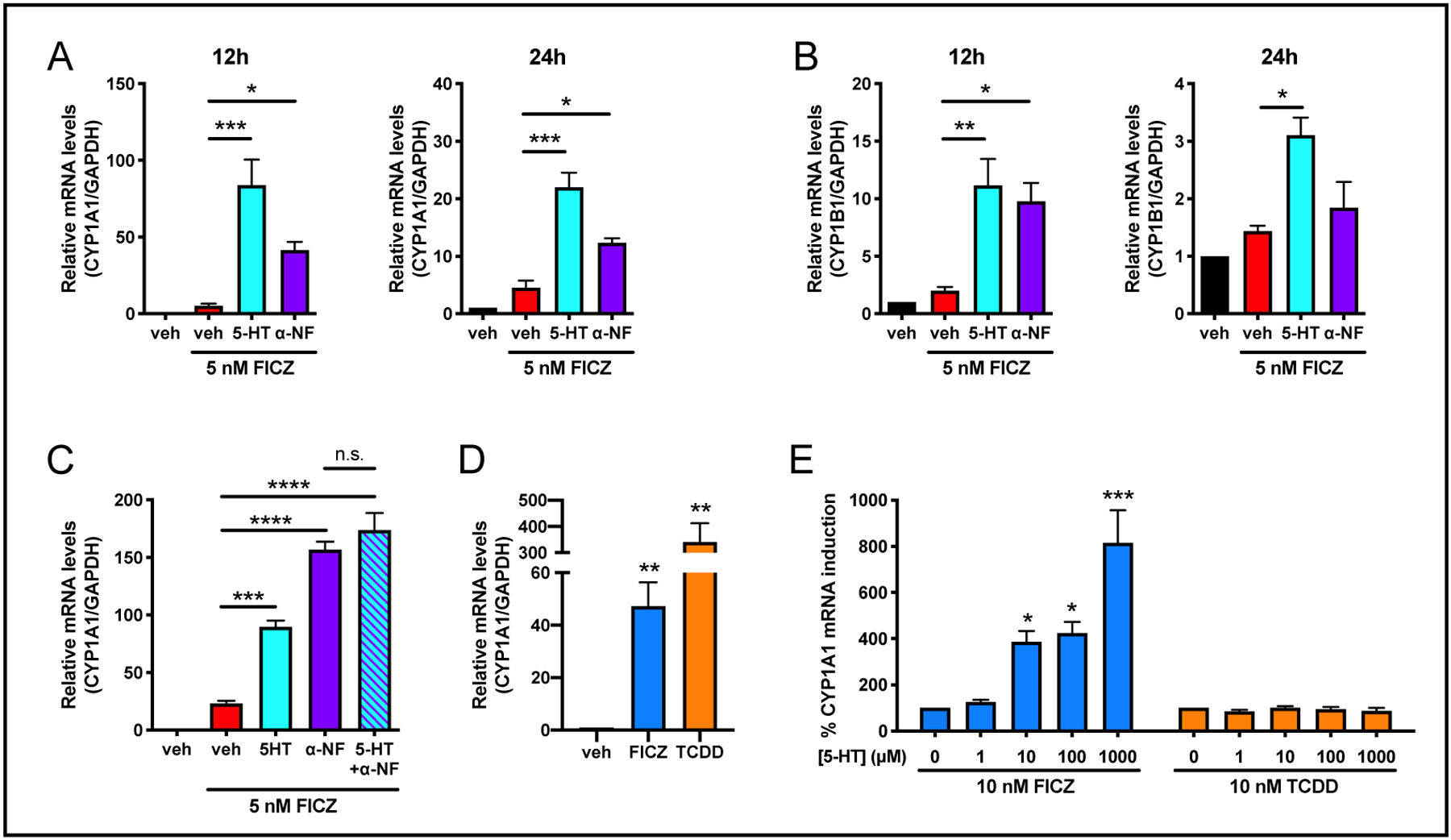
5-HT potentiates induction of AhR target genes by ligands which are CYP1A1 substrates. Caco-2 cells were plated at low density and allowed to differentiate for 10–14 d in medium containing 10% serum before treatments were performed. (A and B) Cells were treated with vehicle (veh) or 5 nM FICZ in the presence of vehicle, 5-HT (10 μM), or α-NF (1 μM) for 12 h or 24 h in TRP-free and serum-free medium. Data represent the relative expression of CYP1A1 (A) or CYP1B1 (B) mRNA quantified by qPCR as compared to time-matched vehicle cells (n = 3). (C) Cells were also treated with vehicle or 5 nM FICZ in the presence of vehicle, 5-HT (5 μM), α-NF (5 μM), or 5-HT + α-NF in combination (both 5 μM) for 16 h in TRP-free and serum-free medium (n = 4). Data analyzed by 1-way ANOVA followed by Tukey’s multiple comparisons test. (D and E) Cells were treated with vehicle (veh), FICZ (10 nM), or TCDD (10 nM) for 24 h in the presence of absence of 5-HT (1 – 1000 μM) in serum-free and TRP-free medium. (D) Relative CYP1A1 mRNA induction after FICZ or TCDD alone is expressed as fold-change over vehicle and (E) CYP1A1 mRNA expression in the presence of FICZ or TCDD with increasing concentrations of 5-HT expressed as percentage of induction without 5-HT. CYP1A1 mRNA was quantified by qPCR (n = 3–4). Data analyzed by Student’s unpaired t-test compared to vehicle treated cells (D) or 1-way ANOVA followed by Dunnett’s multiple comparisons test (E). *P<0.05, **P<0.01, ***P<0.001, ****P<0.0001. n.s. = not significant.

Since 5-HT was able to prevent degradation of FICZ (Fig. 2B), we next examined whether simultaneous addition of 5-HT and FICZ to the TRP free medium will induce transcription of AhR target genes. Caco-2 cells were treated with 5-HT (10 μM) along with FICZ (5 nM). At both 12 h and 24 h, 5-HT was able to potentiate transcription of both *CYP1A1* and *CYP1B1* by FICZ (Fig. 5A and B). A similar pattern was observed with α-NF (1 μM). To confirm that 5-HT and α-NF were acting through the same mechanism, namely CYP1A1 inhibition, Caco-2 cells were treated with FICZ (5 nM) along with 5-HT (5 μM) and α-NF (5 μM) simultaneously for 16 h. There was no further augmentation of *CYP1A1* mRNA induction by 5-HT compared to α-NF alone, suggesting that both molecules work through the same pathway (Fig. 5C).

2, 3,7, 8-Tetrachlorodibenzo-*p*-dioxin (TCDD) is a well-described high affinity AhR ligand that is not a substrate of CYP1A1 [36]. As expected, TCDD treatment (10 nM) dramatically induced CYP1A1 expression in Caco-2 cells after 24 h (Fig. 5D). To examine whether 5-HT is able to augment AhR activation by TCDD in a similar manner as FICZ, Caco-2 cells were treated with either 10 nM FICZ or TCDD along with increasing concentrations of 5-HT (1 – 1000 μM). In accordance with our previous results, 5-HT was able to enhance *CYP1A1*mRNA induction by FICZ (Fig. 5E). However, 5-HT had no effect on *CYP1A1* mRNA induction by TCDD. Of note, many indole-derivatives which are AhR agonists are actually partial agonists, and attenuate induction by the full agonist TCDD [37] (Supplementary Fig. S1). This phenomenon was not observed with 5-HT. These results are consistent with the finding that 5-HT augments AhR activation by AhR ligands which are also CYP1A1-substrates, without affecting activation by ligands which are not metabolized by CYP1A1.

### Activation of AhR by Orally Administered AhR Agonists is Impaired in SERT KO Mice

We previously showed that SERT is necessary for 5-HT dependent activation of AhR both *in vitro* and *in vivo*, presumptively by allowing 5-HT to accumulate intracellularly [18]. SERT KO mice lack high affinity transport of 5-HT into the intracellular compartment [38]. Based upon our *in vitro* findings that 5-HT is interfering with CYP1A1 metabolism of AhR ligands, we hypothesized that SERT KO mice would be able to metabolize exogenously administered AhR ligands more rapidly, leading to impaired activation of AhR by these ligands.

**Fig. 6.**
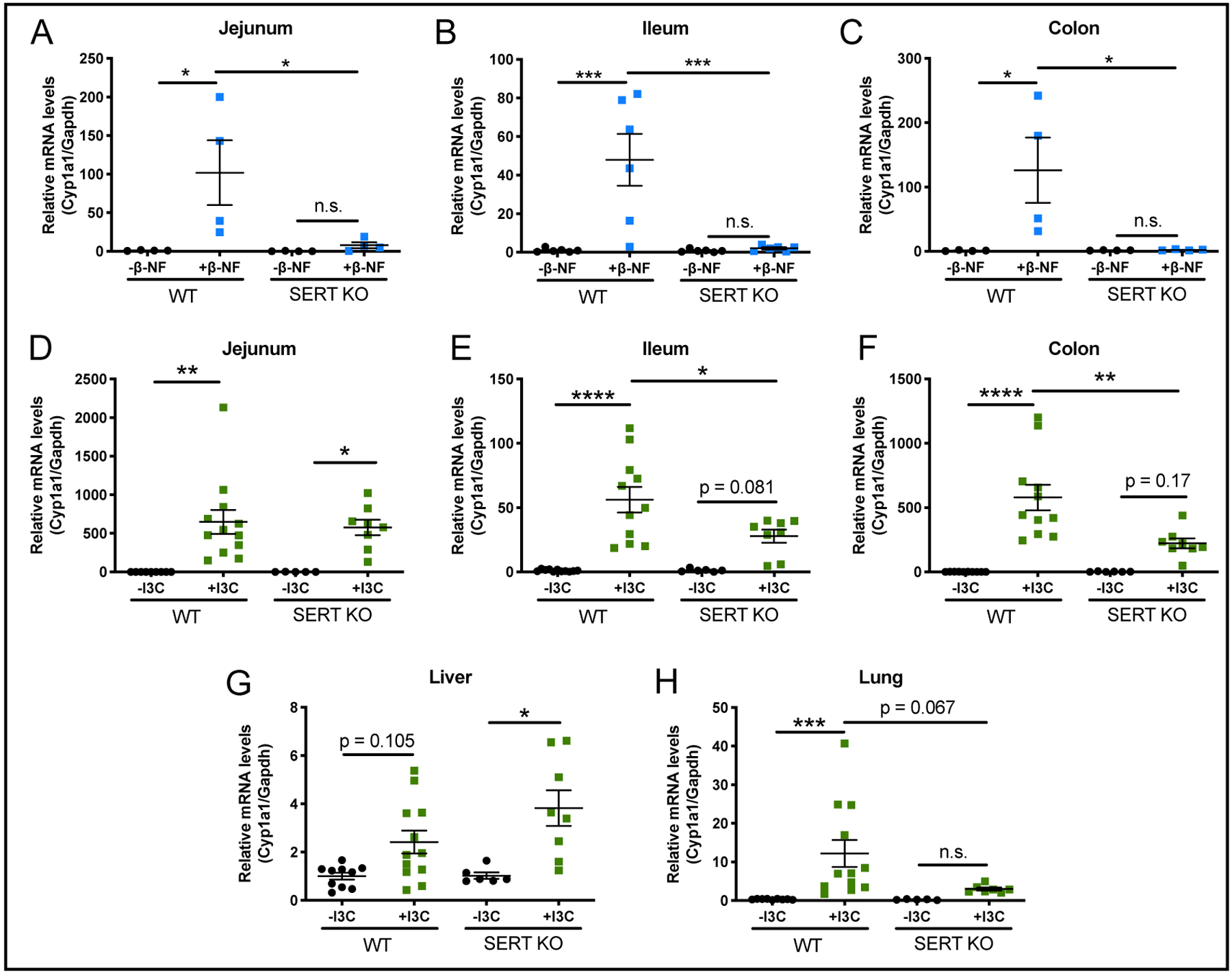
SERT KO mice have impaired AhR activation in response to exogenously administered AhR ligands. (A-C) WT and SERT KO mice were administered β-naphthoflavone (β-NF) by gavage (50 mg/kg) in corn oil once a day for 4 days. Total RNA was isolated from jejunal (A), ileal (C), or colonic (C) mucosal scrapings. Data represent the relative expression of Cyp1a1 mRNA quantified by qPCR (n = 4–6). (D-H) WT and SERT KO mice were placed on an AIN-76A synthetic diet with or without 200 ppm indole-3-carbinol (I3C) for 3 weeks. Total RNA was isolated from jejunal (D), ileal (E), or colonic (F) mucosal scrapings as well as whole liver (G) and lung (H). Data represent the relative expression of Cyp1a1 mRNA quantified by qPCR (n = 6–12). Data analyzed by 2-Way ANOVA followed by Tukey’s multiple comparison’s test. *P<0.05, **P<0.01, ***P<0.001, ****P<0.0001 between groups as indicated. n.s. = not significant.

β-naphthoflavone (β-NF) is a high-affinity synthetic AhR ligand [39] that is a CYP1A1 substrate [40] and has also been shown to decrease the severity of DSS-colitis [4]. In order to determine whether 5-HT is able to affect activation of AhR by AhR ligands *in vivo*, we administered β-NF to WT and SERT KO by gavage (50 mg/kg/day in corn oil, 3 days). In WT mice, β-NF induced intestinal *Cyp1a1* mRNA ~100-fold in jejunum and colon, and ~50 fold in ileum whereas induction in SERT KO mice did not reach significance (Fig. 6A–C).

To complement the β-NF gavage studies, we examined the ability of indole-3-carbinol (I3C) to activate AhR in WT and SERT KO mice. Control mice were given AIN-76A, a synthetic diet devoid of phytochemicals that could potentially activate AhR, and dietary I3C was given as 200 ppm in an AIN-76A background diet for 3 weeks. In jejunum, the I3C diet induced *Cyp1a1* mRNA ~500-fold with no significant difference between WT and SERT KO (Fig. 6D). In ileum and colon, *Cyp1a1* mRNA was induced in WT mice ~50-fold and ~500-fold, respectively, with significantly less induction in SERT KO mice (Fig. 6E and F). Induction of *Cyp1a1* mRNA in liver was modest in both WT and SERT KO mice with a ~2–4-fold increase after the I3C diet as compared to the control diet (Fig. 6G) Intriguingly, *Cyp1a1* mRNA was induced ~12-fold in WT lung with no significant induction in SERT KO lung after the I3C diet (Fig. 6H).

**Fig. 7.**
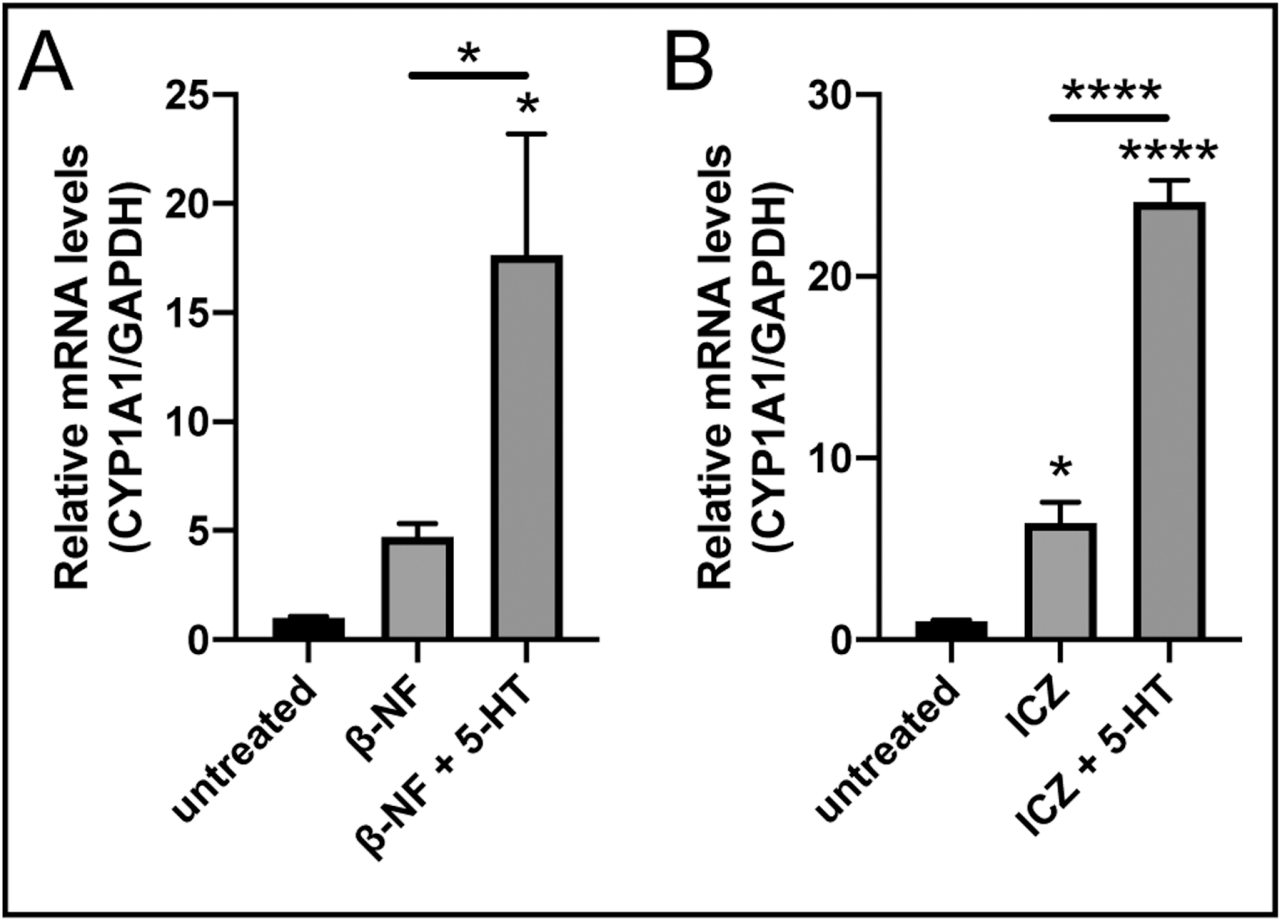
5-HT potentiates CYP1A1 induction by β-NF and ICZ. Caco-2 cells were plated at low density and allowed to differentiate for 10–14 d in medium containing 10% serum before treatments were performed. Cells were treated with 1 μM β-NF (A) or 50 nM ICZ (B) in the presence of 5-HT (10 μM) for 16 h in TRP-free and serum-free medium. Data represent the relative expression of CYP1A1 mRNA quantified by qPCR as compared to untreated cells (n = 4). Data analyzed by 1-way ANOVA followed by Tukey’s multiple comparisons test. *P<0.05, ****P<0.0001 compared to untreated cells or between groups as indicated.

Induction of CYP1A1 mRNA in Caco-2 cells with either β-NF (1 μM) or ICZ (50 nM), the high affinity AhR ligand formed from I3C in stomach acid, in TRP-free medium was enhanced in the presence of 5-HT (10 μM) (Fig. 7). Collectively, these results are consistent with a novel role for 5-HT in modulating the ability of exogenously administered AhR ligands to induce the expression of AhR target genes *in vivo*.

## Discussion

The current study has delineated the molecular mechanism by which AhR is activated by 5-HT. We provide evidence that 5-HT itself is not a ligand for AhR, but rather 5-HT modulatess activation of AhR by its ligands. We demonstrated that 5-HT is a CYP1A1 substrate that competes with AhR ligands for CYP1A1 degradation, preventing ligand deactivation and promoting sustained AhR signaling. Activation of AhR by both endogenous ligands such as FICZ and exogenous ligands such as α-NF were shown to be affected by 5-HT. Therefore, we have established a novel role of 5-HT as an endogenously produced modulator of the AhR signaling pathway.

Inhibition of CYP1A1 enzymatic activity by 5-HT was demonstrated in both a cell-based and in a cell-free system. Co-incubation of Caco-2 cells with the CYP1A1 substrate Luc-CEE showed that 5-HT dramatically decreases its metabolism at only 5 μM. This concentration is nearly identical to the local steady state 5-HT concentration at the surface of the murine ileal mucosa, providing evidence that inhibition can occur at physiologic concentrations of 5-HT [41]. These data were corroborated by showing directly that 5-HT interfered with FICZ degradation in Caco-2 cells also at 5 μM. When metabolism of Luc-CEE using a recombinant system was assessed, it was found that both 5-HT and TRYPT similarly interfered with CYP1A1 activity with slightly less potency than α-NF. The lack of inhibition by 5-HIAA and IAA provide further evidence that the inhibition observed is specific to 5-HT.

The observation of metabolism of 5-HT by CYP1A1, which we found to be central to the involvement of 5-HT in the AhR signaling pathway, has not been reported previously. However, it was found that the structurally-related melatonin is hydroxylated at the 6-position by CYP1A1, CYP1A2, and CYP1B1 [42]. Therefore, we can hypothesize that the major product of CYP1A1 metabolism of 5-HT is 5, 6-dihydroxytryptamine (5, 6-DHT]. While it is not detected in brain at baseline, endogenously formed 5, 6-DHT is detected in rat brain upon administration of monoamine oxidase inhibitors [43, 44]. It is very likely that 5-HT is able to interfere with CYP1A2 and CYP1B1 activity since the CYP1 family of enzymes have significant substrate overlap. Interestingly, a previous study provided evidence that 5-HT interferes with CYP1A2 metabolism by competing for phenacetin *O*-de-ethylation activity in liver microsomes [45].

Key evidence which suggests that 5-HT is not a ligand for AhR is the lack of induction in TRP-free medium, devoid of the low levels of FICZ found within routine cell culture medium [15, 35]. Many studies exist which assume that a compound which induces CYP1A1 mRNA or AhR reporter activity *in vitro* is an AhR ligand [46, 47]. Such studies should be complemented with experiments showing AhR activation in a system without AhR ligands to rule out indirect activation by inhibiting ligand clearance by CYP1A1 or otherwise. Addition of recrystallized TRP immediately prior to treatment did not alter the results, an important control since TRP depletion may affect cell viability or other cellular processes. The conclusion that 5-HT is not an AhR ligand is consistent with a prior study which performed an electrophoretic mobility shift assay for AhR binding and found that 5-HT failed to induce an AhR-dependent shift [48].

Transcription of AhR target genes by low concentrations of FICZ in TRP-free medium was significantly enhanced by co-treatment with 5-HT. This was true at both 12 h and 24 h time points for both *CYP1A1* and *CYP1B1* mRNA. However, induction was much stronger at 12 h than 24 h. This might be due to excessive degradation of FICZ after 24 h, even in the presence of CYP1A1 inhibitors. Alternatively, AhR receptor levels could have decreased due to proteasomal degradation of the receptor following excessive activation [49]. Interestingly, only FICZ in the presence of 5-HT was able to induce CYP1B1 induction after 24 h. This provides evidence that transcription of some target genes may only occur in the setting of CYP1A1 inhibition under certain conditions, underscoring the importance of CYP1A1-mediated ligand clearance in controlling AhR activation.

While SERT KO mice were utilized in our studies as a model with decreased intracellular 5-HT, other implications of decreased SERT must be considered while interpreting our data. First, it might be predicted that SERT deficiency may decrease intracellular transport of other molecules which may affect CYP1A1 expression [50]. However, we have shown previously that SERT overexpression does not affect CYP1A1 induction by several TRP-derived AhR ligands which suggests that the ability of SERT to modulate AhR activation is specific to 5-HT [18]. Additionally, there may be a concern that altered intestinal transit resulting from SERT deletion could influence activation of AhR by orally administered AhR ligands by affecting their absorption. While SERT KO mice do exhibit altered GI motility patterns, there is no overall change in GI transit time [38, 51]. Lastly, one might speculate that other 5-HT transporters may compensate for the absence of SERT in IECs. While several organic cation transporters (OCTs) are expressed in IECs are able to transport 5-HT with low affinity, uptake of radiolabeled 5-HT by intestinal mucosal tissues is observed only in WT mice but not in SERT KO [38]. Thus, OCTs on IECs likely prevent accumulation of extracellular 5-HT to toxic levels, but do not participate in normal clearance of 5-HT.

The extent of the differences in intestinal CYP1A1 induction *in vivo* was dependent upon the ligand administered. The synthetic ligand β-NF was administered by gavage 3 consecutive days prior to sacrifice. In all segments of the intestine, CYP1A1 mRNA induction in SERT KO mice did not reach significance, whereas WT mice exhibited variable, yet significant, induction. Of note, the half-life of CYP1A1 mRNA was found to be ~2.4 h [52], which likely contributes to the high variability in CYP1A1 induction. While WT and SERT KO mice displayed differential AhR activation in response to the I3C diet, the differences were not as dramatic as was observed with β-NF gavage. In jejunum, there was no difference in CYP1A1 induction between WT and SERT KO mice. However, significant differences in CYP1A1 induction were observed in both ileum and colon. One of the reasons for these differences could be that I3C was given *ab libitum* for 3 weeks whereas β-NF was given as gavage for 3 days. Continuous activation of AhR during feeding could lead to more sustained CYP1A1 mRNA levels as opposed to the staggered activation by gavage. Another reason could be the nature of the ligands themselves. β-NF is an excellent CYP1A1 substrate [40] whereas I3C gets converted into ICZ and DIM which have variable rates of metabolism by CYP1A1 [53, 54].

While the modest induction of CYP1A1 in liver after the I3C diet was not different between WT and SERT KO mice, there was an attenuation of CYP1A1 induction in the lungs of SERT KO mice. While there are a few possible mechanisms which may explain this result, it is likely that ingested ligands reaching the circulation eventually made their way to the lungs. Lung tissue is known to express many components of serotonergic machinery, including SERT [25]. The lack of SERT within the lung epithelium in SERT KO mice could have led to impaired CYP1A1 induction due to enhanced ligand clearance.

## Conclusion

In conclusion, we have found that 5-HT is a CYP1A1 substrate that competes with AhR ligands for CYP1A1 degradation, preventing ligand deactivation and promoting sustained AhR signaling. This knowledge enhances our understanding of both intrinsic regulation of AhR activation and 5-HT metabolism. Further, these findings also provide novel insight into how the serotonergic pathway may be exploited pharmacologically to modulate AhR signaling in diseases with altered AhR activation in the intestine.

## Supplementary Material

Supplemental material
