# Supplementary material for: Serotonin Modulates AhR Activation by Interfering with CYP1A1-Mediated Clearance of AhR Ligands
Source: Cell Physiol Biochem. Author manuscript; Available in PMC 2020 Mar 2. (PMC7050772; doi:10.33594/000000209)
Supplement: Supplemental material [file NIHMS1558865-supplement-Supplemental_material.pdf]

Supplemental Material

Serotonin Modulates AhR Activation by Interfering with CYP1A1-Mediated Clearance of AhR Ligands

Christopher R. Manzella[a] Max Ackerman[b] Megha Singhal[b] Alexander L. Ticho[a] Justin Ceh[b]
Waddah A. Alrefai[b,c] Seema Saksena[b,c] Pradeep K. Dudeja[b,c]
Ravinder K. Gill[b]

[a]Department of Physiology & Biophysics, University of Illinois at Chicago, Chicago, IL, USA, [b]Division of Gastroenterology & Hepatology, University of Illinois at Chicago, Chicago, IL, USA, [c]Jesse Brown VA Medical Center, Chicago, IL, USA

Supplemental Information

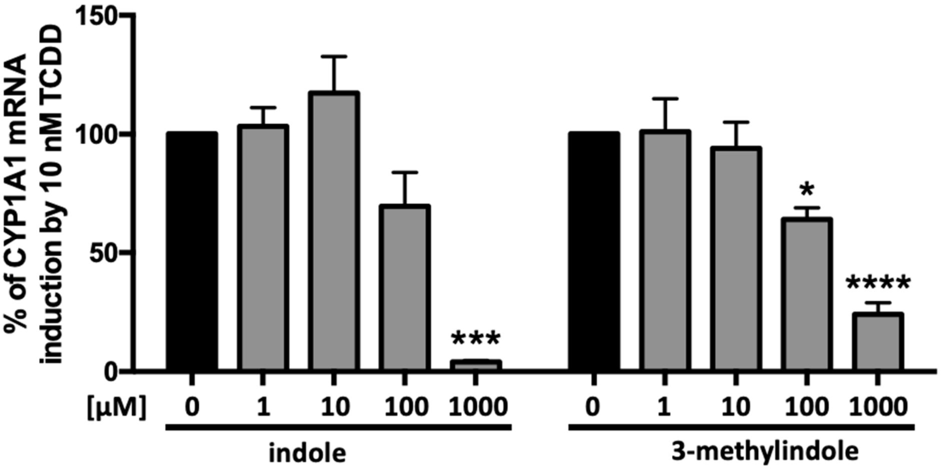

Figure S1: Tryptophan-derived partial AhR agonists block CYP1A1 induction by TCDD. Caco-2 cells were plated at low density and allowed to differentiate for 10-14 d in medium containing 10% serum before treatments were performed. Cells were treated with vehicle, indole, or 3-methylindole (both 1 – 1000 µM) in the presence of 10 nM TCDD in serum-free and TRP-free medium. Results expressed as percentage *CYP1A1* mRNA induction without indole or 3-methylindole. *CYP1A1* mRNA was quantified by qPCR. Data analyzed by 1-way ANOVA followed by Dunnett's multiple comparisons test. *$P < 0.05$, ***$P < 0.001$, ****$P < 0.0001$ as compared to cells treated in the absence of indole or 3-methylindole.

Table S1: Gene-specific primers used for Real-Time RT-PCR.

Gene	Species	Accession No.	Primer sequence (5'3')
CYP1A1	Human	NM_000499	F: TCGGCCACGGAGTTTCTTC<br>R: GGTCAGCATGTGCCCAATCA
GAPDH	Human	NM_001101.3	F: GAAATCCCATCACCATCTTCC<br>R: AAATGAGCCCCAGCCTTCT
CYP1B1	Human	NM_000104	F: TGCCTGTCACTATTCCTCATGCCA<br>R: ATCAAAGTTCTCCGGGTTAGGCCA
Cyp1a1	Mouse	NM_001136059	F: GGGTTTGACACAGTCACAACT<br>R: GGGACGAAGGATGAATGCCG
Gapdh	Mouse	NM_008084	F: TGTGTCCGTCGTGGATCTGA<br>R: CCTGCTTCACCACCTTCTTGAT

Table S2: LC method 1.

Time (min)	A (%)	B (%)
-3	97	3
0	97	3
0.5	97	3
1	70	30
2	40	60
2.25	40	60
2.5	97	3

Table S3: LC method 2.

Time (min)	A (%)	B (%)
-3	97	3
0	97	3
0.5	97	3
1	70	30
2	40	60
3	40	60
3.3	5	95
5	5	95
5.2	97	3
6	97	3

Table S4: LC method 3.

**Time (min)**	**A (%)**	**B (%)**
-2.5	50	50
0	50	50
1.5	0	100
3.5	0	100
3.7	50	50

Table S5: MRM parameters for LC-MS/MS measurements.

**Analyte**	**Q1 mass (*m*/*z*)**	**Q3 mass (*m*/*z*)**	**DP (V)**	**CE (V)**
L-tryptophan	205	118	57	36
L-tryptophan	205	188	57	14
serotonin	177	160	54	16
serotonin	177	115	54	37
5-hydroxyindoleacetic acid	192	146	18	28
FICZ	284	255	-148	-34
